# Double Cross-Leg Reverse Sural Flap for Coverage of an Extensive Single-Leg Soft-Tissue Defect: A Case Report

**DOI:** 10.7759/cureus.108020

**Published:** 2026-04-30

**Authors:** Edgar A Flores García, Saúl Ibarra Vázquez, Jennifer Navarro Morales, Francisco J Rivera Vazquez, Abigail Diaz Alba, Gerardo A Mancillas, Gadiel E Pérez Morales

**Affiliations:** 1 Surgery, Hospital General Nuevo de Gómez Palacio, Gómez Palacio, MEX; 2 Plastic and Reconstructive Surgery, Hospital General Nuevo de Gómez Palacio, Gómez Palacio, MEX; 3 Surgery, Hospital General de Lerdo, Lerdo, MEX

**Keywords:** reverse sural artery fasciocutaneous flaps, reverse sural flap, sural fasciocutaneous flap, sural flap, sural nerve

## Abstract

Extensive soft-tissue defects of the distal lower extremity following high-energy trauma represent a major reconstructive challenge, particularly when involving the heel and ankle. The reverse sural flap is a well-established option for distal leg and foot coverage; however, its dimensions are traditionally limited, and large defects often require free tissue transfer. We report the case of a 23-year-old male who sustained a high-speed motorcycle accident resulting in an extensive right heel and ankle soft-tissue defect with exposed calcaneus, associated with calcaneal fracture, subtalar dislocation, and talar dislocation. After fracture reduction and serial surgical debridements, a circular defect measuring approximately 18 cm in diameter persisted. Due to the absence of a microsurgical service, bilateral crossed reverse sural fasciocutaneous flaps were performed to achieve adequate coverage. Pedicles measuring approximately 14 cm were transposed in a cross-leg fashion, and both lower limbs were temporarily stabilized with transosseous tibial fixation for three weeks. Following pedicle division, both flaps demonstrated full vascular autonomy without partial or total necrosis. Donor sites were covered with split-thickness skin grafts harvested from the posterior thighs. At nearly one year of follow-up, the patient achieved partial ambulation with stable soft-tissue coverage, preserved protective sensitivity, and satisfactory scar maturation. Bilateral crossed reverse sural flaps may represent a viable limb-salvage strategy for massive unilateral heel and ankle defects when microsurgical reconstruction is not feasible, expanding the traditional indications of the reverse sural flap in complex post-traumatic injuries.

## Introduction

High-energy lower extremity trauma, particularly following motorcycle accidents, frequently results in complex soft-tissue defects with exposure of bone, tendons, or orthopedic hardware, posing significant reconstructive challenges [1]. Early and adequate soft-tissue coverage is essential to reduce infection rates, improve wound healing, and preserve limb function [2].

The reverse sural flap has become a widely accepted and reliable reconstructive option for defects involving the distal third of the leg, ankle, and foot [3]. Based on the vascular axis accompanying the sural nerve and the septocutaneous perforators of the peroneal artery, this flap provides a technically straightforward alternative to free tissue transfer [4]. Its consistent anatomy, relative ease of elevation, and acceptable donor-site morbidity have contributed to its widespread use in lower limb reconstruction [3,4].

Despite its versatility, the reverse sural flap is traditionally employed as a unilateral reconstructive option, and its dimensions are limited by vascular reliability and arc of rotation [5]. In cases of extensive unilateral soft-tissue loss secondary to high-energy trauma, a single flap may be insufficient to achieve adequate coverage.

Reports describing the simultaneous use of bilateral crossed reverse sural flaps to reconstruct a single large unilateral defect are exceedingly rare in the literature [6]. We present the case of a patient with a massive post-traumatic lower limb soft-tissue defect following a motorcycle accident successfully managed with bilateral crossed reverse sural flaps, highlighting this technique as a potential limb-salvage strategy in selected patients.

## Case presentation

A 23-year-old male was admitted to the emergency department after sustaining a high-speed motorcycle accident. On initial evaluation, he presented with an extensive soft-tissue injury involving the right foot and ankle, characterized by a large area of devitalized tissue with exposure of the calcaneus and surrounding soft tissues. Associated injuries included a calcaneal fracture, subtalar dislocation, and talar dislocation.

The trauma surgery team performed emergent reduction of the fractures and dislocations, along with thorough surgical debridement. Initial trauma assessment, including laboratory studies, chest and abdominal radiographs, and a focused assessment with sonography for trauma, revealed no additional injuries. The patient remained hemodynamically stable.

Plastic surgery was consulted for evaluation of the extensive soft-tissue defect and reconstructive planning. Over the following seven days, serial wound care and repeated surgical debridements were performed to obtain a clean and viable wound bed.

After adequate preparation, a circular soft- tissue defect measuring approximately 18 cm in diameter persisted, involving the heel and ankle region, with exposed calcaneus. The size and location of the defect precluded primary closure or coverage with a single local flap.

**Figure 1:**
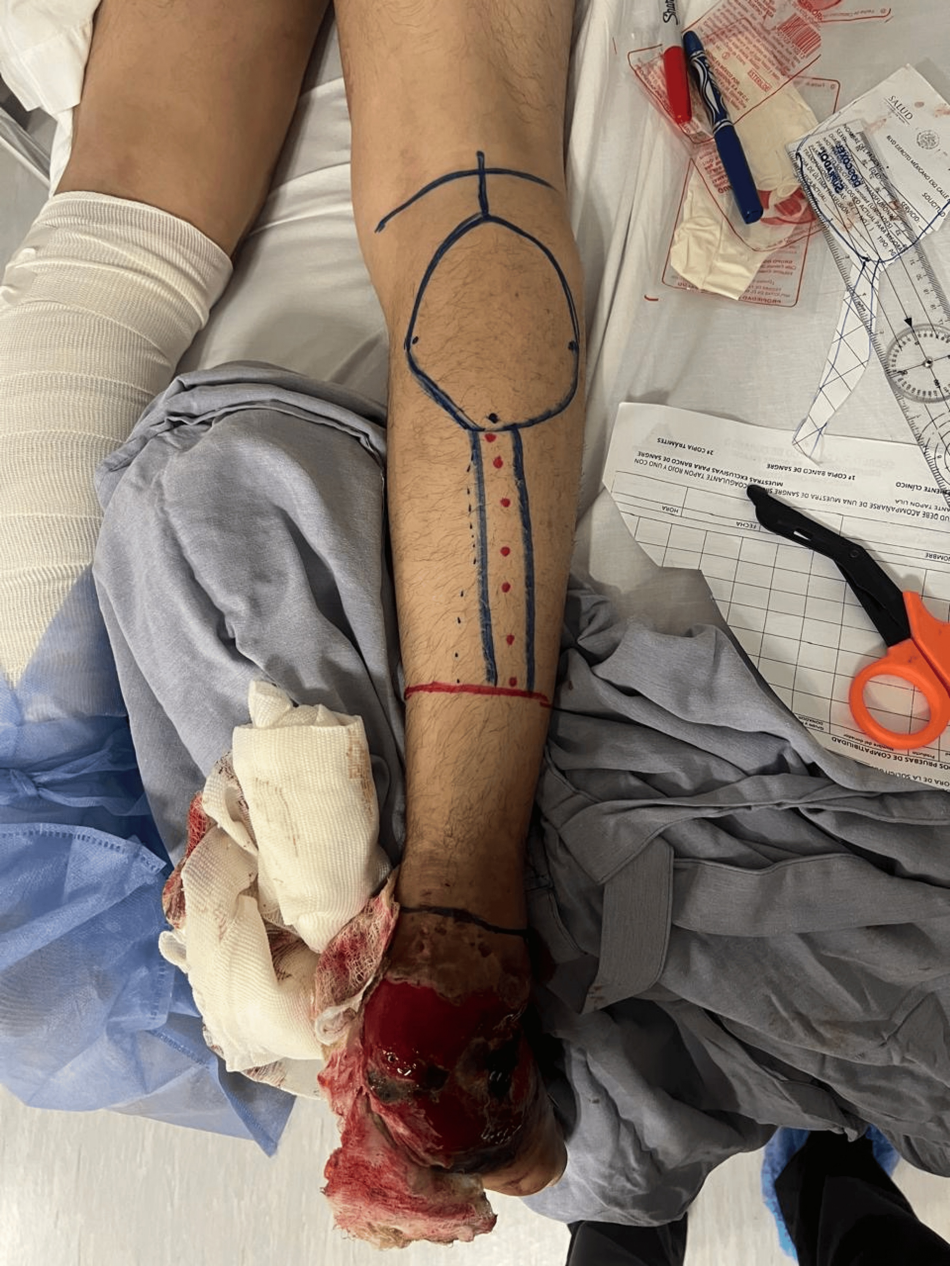
Preoperative planning for a distally based sural artery flap (retrograde sural fasciocutaneous flap) intended for the reconstruction of a large soft-tissue defect in the hindfoot and heel region. Markings indicate the flap island on the proximal/middle third of the posterior leg, the vascular pedicle axis corresponding to the sural nerve and midline artery, and the pivot point located approximately 5 cm superior to the lateral malleolus.

Given the magnitude of the defect and the absence of a microsurgical service at our institution, a decision was made to proceed with bilateral crossed reverse sural fasciocutaneous flaps as a limb-salvage strategy (Figure 1).

Under general anesthesia, reverse sural flaps were designed on the posterior aspect of both lower extremities. Each flap was elevated, including the sural nerve, lesser saphenous vein, and deep fascia. The pedicles measured approximately 14 cm in length to allow an adequate arc of rotation. Both flaps were transposed in a cross-leg fashion to cover the extensive unilateral defect of the right lower extremity.

**Figure 2:**
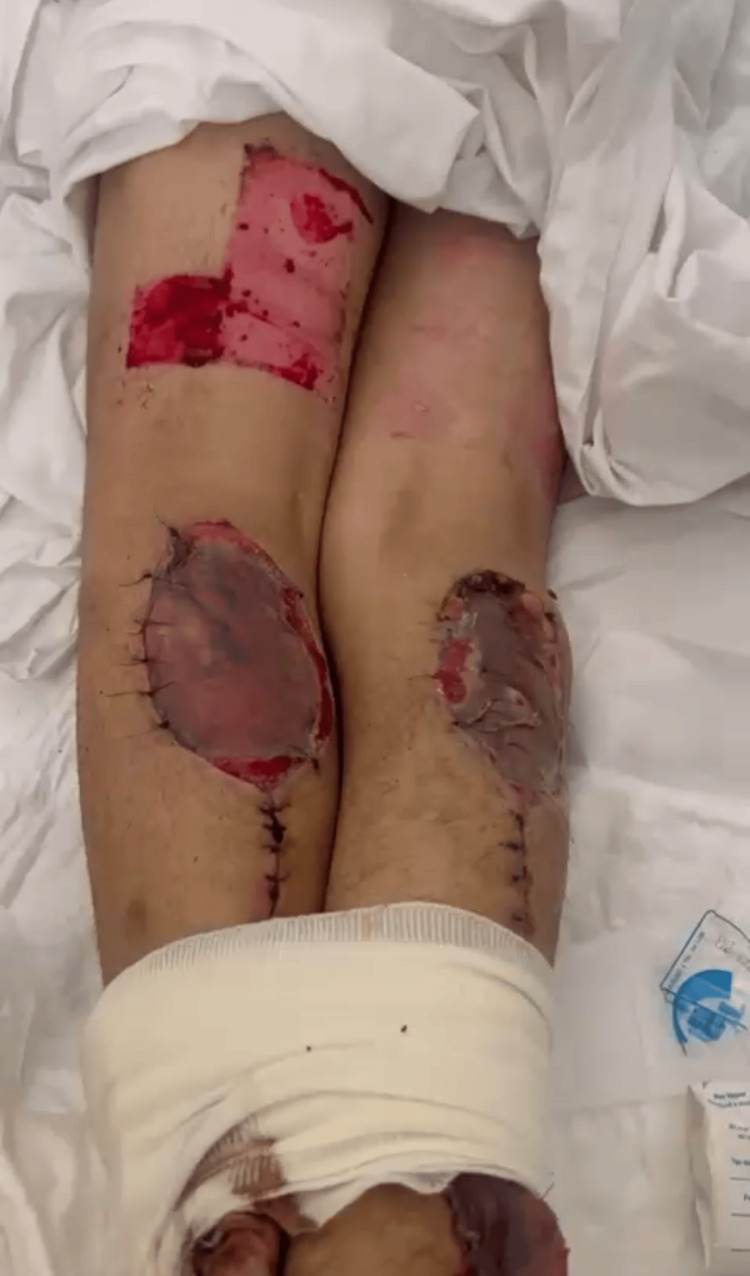
Donor-site assessment and supplementary coverage. A clinical view of both lower extremities showing the sural flap donor site on the ipsilateral leg, managed with primary closure using continuous sutures. Split-thickness skin graft donor sites are visible on the thigh, harvested for secondary defect coverage. Compressive bandaging on the contralateral limb and the patient’s clinical stability during the postoperative period are noted.

To protect the pedicles and minimize movement, both tibias were temporarily stabilized using transosseous pins, maintaining cross-leg immobilization for three weeks. Donor sites were covered with split-thickness skin grafts harvested from the posterior aspect of each thigh using a dermatome to ensure appropriate graft thickness (Figure 2).

**Figure 3:**
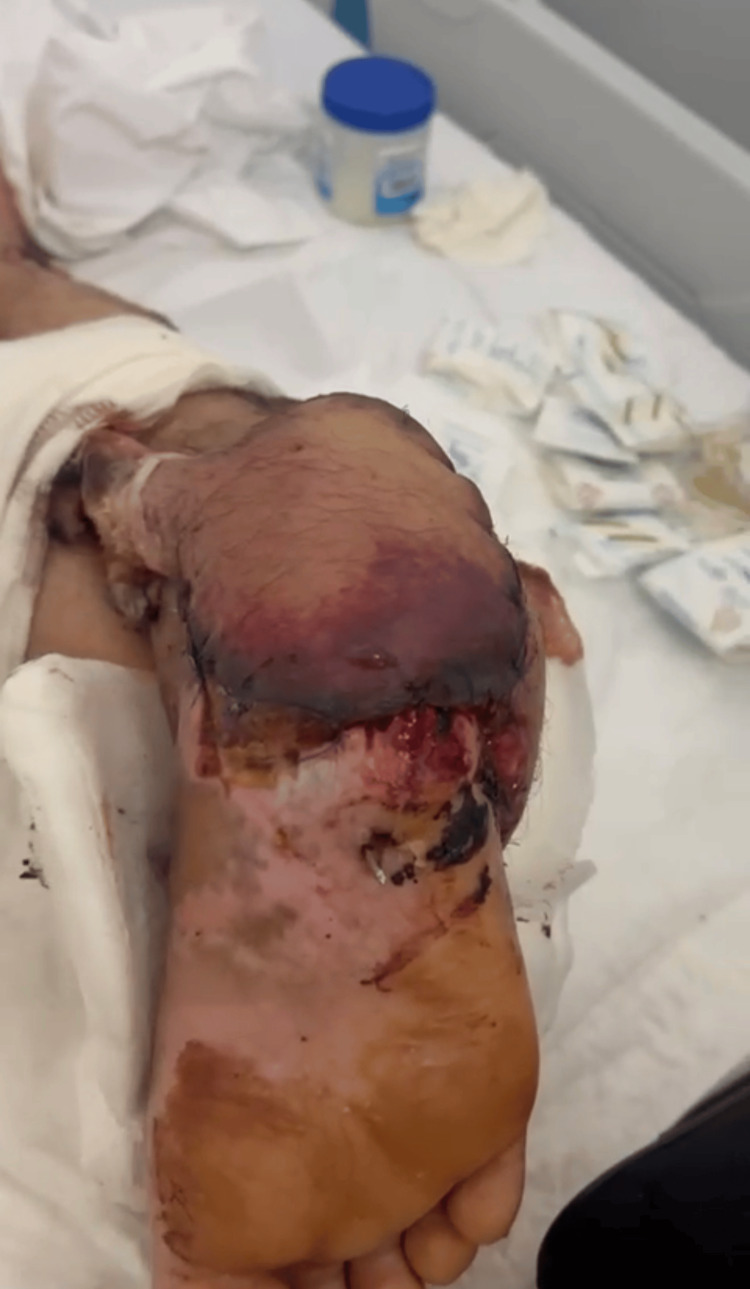
Lateral view during the early postoperative period, showing the pedicle rotation at the distal pivot point and proper skin island apposition to the recipient bed with interrupted sutures. Clinical follow-up demonstrating signs of venous congestion and violaceous discoloration at the distal margins of the flap, with surrounding granulation tissue. Percutaneous K-wire fixation for skeletal stabilization remains in place.

At three weeks postoperatively, pedicle division was performed. A transient color change was observed immediately after sectioning; however, capillary refill and tissue tone progressively normalized, confirming adequate vascular autonomy of both flaps. A small area of partial flap necrosis was observed; however, it was self-limited, with no progression, and resolved with conservative management without compromising overall flap viability (Figure 3).

**Figure 4:**
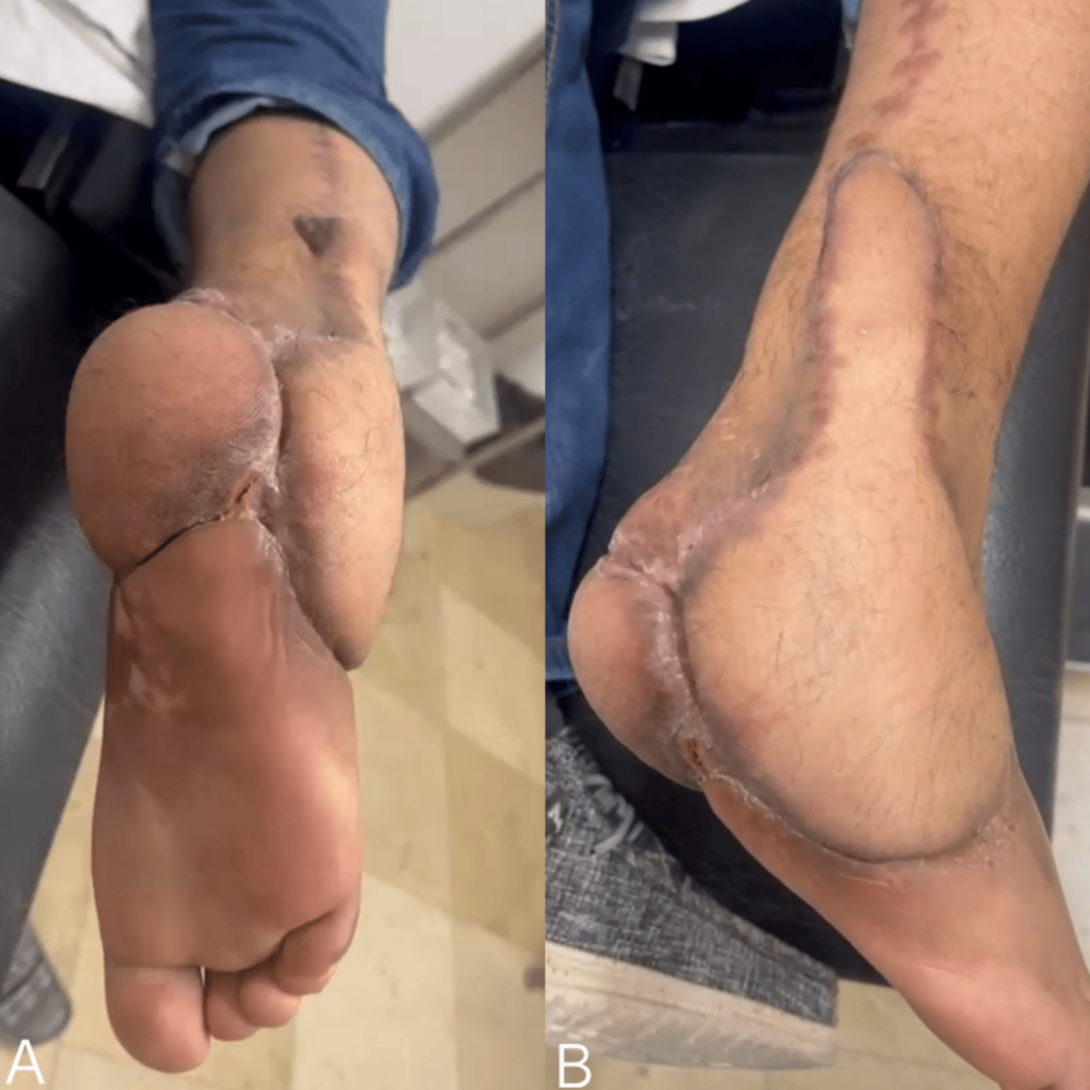
Clinical evolution following complex hindfoot reconstruction. (A) Plantar view of the reconstructed heel, demonstrating successful integration of the transferred tissue into the weight-bearing area. (B) Posterolateral perspective illustrating the flap volume and the integrity of the underlying Achilles tendon region. Sutures have been removed, and the surgical margins show satisfactory healing without evidence of dehiscence or ischemia.

The patient was followed jointly by the plastic surgery and trauma teams. At nearly one year of follow-up, the patient achieved partial ambulation with stable soft-tissue coverage. The flaps demonstrated good contour, satisfactory scar maturation, preserved protective sensitivity, and complete wound healing without evidence of breakdown or infection (Figure 4).

Written informed consent was obtained from the patient for publication of this case report and accompanying images.

## Discussion

Reconstruction of extensive distal lower extremity defects, particularly those involving the heel and ankle, remains a significant challenge in reconstructive surgery. The heel requires durable, well-vascularized coverage capable of withstanding weight-bearing forces, while the ankle region presents limited local tissue availability and restricted flap mobility [6,7].

The reverse sural flap has become a well-established option for coverage of distal third leg, ankle, and heel defects. Its anatomical basis has been well described, relying on the vascular axis accompanying the sural nerve and the lesser saphenous vein, with consistent distal perforators from the peroneal artery [1-3]. Since its clinical popularization as a distally based fasciocutaneous flap [4,8], it has gained widespread acceptance due to its technical simplicity and avoidance of microsurgical anastomosis.

Multiple series have demonstrated its effectiveness in distal lower limb reconstruction [6,7]. However, flap dimensions remain limited by vascular reliability, particularly in large defects. Complication analyses have shown that increasing flap size and arc of rotation are associated with higher risks of venous congestion and partial necrosis [5]. These limitations make reconstruction of massive heel defects particularly challenging when relying on a single reverse sural flap.

In the present case, the defect measured approximately 18 cm in diameter (approximately 254 cm²), involving both the heel and ankle with exposed calcaneus. Based on the available literature, coverage of a defect of this magnitude would exceed the safe dimensions typically described for a unilateral reverse sural flap [5,8]. Although free tissue transfer is often considered the standard approach for extensive lower extremity defects, especially in high-energy trauma, this option was not available in our institution.

Historically, distally based fasciocutaneous flaps from the sural region evolved as reliable alternatives when microsurgical reconstruction was not feasible [9,10]. Comparative studies have also evaluated sural flaps against other regional options, confirming their versatility but acknowledging their size-related limitations [9,10]. In this context, we opted to increase the reconstructive surface area by employing bilateral crossed reverse sural flaps.

The use of two fasciocutaneous flaps allowed adequate coverage of the large circular defect while maintaining pedicle lengths of approximately 14 cm to ensure safe rotation. Temporary tibial fixation provided stable immobilization during the three-week integration period. Despite a transient color change observed after pedicle division, both flaps achieved full vascular autonomy without partial or total necrosis.

At nearly one year of follow-up, the patient demonstrated stable coverage, preserved protective sensitivity, and partial ambulation. This favorable outcome suggests that bilateral crossed reverse sural flaps may represent a viable limb-salvage alternative in selected patients with massive unilateral heel and ankle defects when microsurgical options are unavailable.

While cross-leg techniques require prolonged immobilization and careful postoperative monitoring, this case expands the potential reconstructive applications of the reverse sural flap beyond its traditionally described unilateral limits.

## Conclusions

Bilateral crossed reverse sural flaps can represent a reliable limb-salvage alternative for massive unilateral heel and ankle defects following high-energy trauma when free tissue transfer is not available. By combining two fasciocutaneous flaps, it is possible to safely expand the reconstructive surface area while maintaining adequate vascular reliability. Although prolonged immobilization is required, this approach may extend the traditional indications of the reverse sural flap and provide durable, functional soft-tissue coverage in complex distal lower extremity injuries.
